# Nanofibrous Scaffolds Support a 3D *in vitro* Permeability Model of the Human Intestinal Epithelium

**DOI:** 10.3389/fphar.2019.00456

**Published:** 2019-05-10

**Authors:** Jamie D. Patient, Hadi Hajiali, Kate Harris, Bertil Abrahamsson, Christer Tannergren, Lisa J. White, Amir M. Ghaemmaghami, Philip M. Williams, Clive J. Roberts, Felicity R. A. J. Rose

**Affiliations:** ^1^School of Pharmacy, University of Nottingham, Nottingham, United Kingdom; ^2^AstraZeneca, Macclesfield, United Kingdom; ^3^AstraZeneca, Mölndal, Sweden; ^4^School of Life Sciences, Queen’s Medical Centre, University of Nottingham, Nottingham, United Kingdom

**Keywords:** Caco-2 cell line, *in vitro* model, permeability, barrier integrity, electrospun scaffolds

## Abstract

Advances in drug research not only depend on high throughput screening to evaluate large numbers of lead compounds but also on the development of *in vitro* models which can simulate human tissues in terms of drug permeability and functions. Potential failures, such as poor permeability or interaction with efflux drug transporters, can be identified in epithelial Caco-2 monolayer models and can impact a drug candidate’s progression onto the next stages of the drug development process. Whilst monolayer models demonstrate reasonably good prediction of *in vivo* permeability for some compounds, more developed *in vitro* tools are needed to assess new entities that enable closer *in vivo in vitro* correlation. In this study, an *in vitro* model of the human intestinal epithelium was developed by utilizing nanofibers, fabricated using electrospinning, to mimic the structure of the basement membrane. We assessed Caco-2 cell response to these materials and investigated the physiological properties of these cells cultured on the fibrous supports, focusing on barrier integrity and drug-permeability properties. The obtained data illustrate that 2D Caco-2 Transwell^®^ cultures exhibit artificially high trans-epithelial electrical resistance (TEER) compared to cells cultured on the 3D nanofibrous scaffolds which show TEER values similar to *ex vivo* porcine tissue (also measured in this study). Furthermore, our results demonstrate that the 3D nanofibrous scaffolds influence the barrier integrity of the Caco-2 monolayer to confer drug-absorption properties that more closely mimic native gut tissue particularly for studying passive epithelial transport. We propose that this 3D model is a suitable *in vitro* model for investigating drug absorption and intestinal metabolism.

## Introduction

Monolayers of cultured epithelial cells are believed to be effective *in vitro* tools to assess the permeation of drugs across epithelial barriers and to probe transporter affinity during the drug development process (Balimane et al., 2000; Balimane and Chong, 2005; Volpe, 2010); as such, they are commonly used for this purpose. Caco-2 cells, originally isolated from a colon adenocarcinoma, develop some of the morphological and functional characteristics when cultured *in vitro* which are akin to absorptive enterocytes found in the small intestine *in vivo*. These characteristics include cellular polarization, apical microvilli and cell-cell tight junctions, and the expression of some of the enzymes and transporters relevant to understanding drug disposition at epithelial barriers. For these reasons, they have been extensively used for the assessment of drug permeability and are a mainstay of both academic and industrial pharmaceutical research (Shah et al., 2016; Yi et al., 2017). In particular, monolayers of cultured human epithelial cells, such as Caco-2, are used in the application of biowaivers (*in vivo* bioavailability studies not considered necessary for product approval), which can reduce the cost and time it takes to develop new medicines (Artursson and Karlsson, 1991; Hubatsch et al., 2007; CDER/FDA, 2015).

Although monolayers of Caco-2 cells provide a simple platform for *in vitro* drug development, it is believed that they still fall short of accurately representing the intestinal epithelium *in vivo*. In particular, the epithelial barrier of cultured Caco-2 cells has been demonstrated to exhibit much higher trans-epithelial electrical resistance (TEER) compared to gut excised tissue segments (Artursson et al., 1993). Furthermore, the prediction of drug absorption kinetics by this model has been reported to be inaccurate (Yi et al., 2017). In fact, it has been shown that poorly permeating compounds, some actively transported drugs, and low molecular weight hydrophilic compounds do not correlate in Caco-2 permeability studies compared to human absorption observed *in vivo* (Balimane et al., 2006). For instance, polyethylene glycols demonstrate almost a 100-fold lower permeability in Caco-2 monolayers when compared to human *in vivo* (Artursson et al., 1993, 2001). In addition, the expression level of relevant transporter proteins (glucose transporter 1, OATP4A1, MRP2, MRP1, and OATP2B1) was significantly higher (3- to 130-fold) in Caco-2 cells compared to human, and all examined CYPs demonstrated lower gene expression in Caco-2 cells compared to human; for instance, difference for CYP3A4 was 871-fold lower expression in Caco-2 cells (Vaessen et al., 2017).

The lack of anatomical and physiological cues that cells experience *in vitro* is one of the foremost reasons for the differences observed between *in vitro* and *in vivo* datasets and furthermore, there is variation in lab-to-lab monolayer Caco-2 permeability results (Lee et al., 2017). For epithelial cell models, the inclusion of additional cell types commonly found in the intestinal mucosa is one approach which enables the reconstitution of tissue-like functions *in vitro*. This includes the use of mucous producing goblet-like cells, such as HT-29/HT-29 MTX, endothelial, immune and stromal cell types that collectively contribute to the dynamic role of the epithelial barrier (Volpe, 2010; Kim et al., 2012; Harrington et al., 2014; Shubber et al., 2015). Other strategies include the introduction of physical and chemical stimuli, usually achieved through biological scaffolds, which include bioactive chemistries or structural cues to modulate cell behavior (Schwarzbauer, 1999; Vllasaliu et al., 2014). Within epithelial tissues, the underlying extracellular matrix (ECM) of the basement membrane has been shown to be significant in the function and maintenance of the epithelium (Schwarzbauer, 1999; Tanjore and Kalluri, 2006).

The aim of this study was to develop a simple, yet more *in vivo*-like 3D *in vitro* model of the human intestinal epithelium. We employed electrospinning to generate porous, fibrous polymeric culture supports that mimic the structure of the basement membrane (Hajiali et al., 2011, 2015, 2018) providing a more realistic environmental condition for cell culture (Morris et al., 2014a; Bischel et al., 2016). We show an assessment of Caco2 response to these materials and investigated the physiological properties of these cells cultured on these fibrous supports, focusing on barrier integrity and drug-permeability properties.

## Materials and Methods

### Fabrication and Surface Treatment of Nanofibrous Scaffolds

Polyethylene terephthalate (PET) is a widely used material in cell culture products including Transwell^®^ inserts. It exhibits high resistance to organic solvents, is durable, low cost, and relatively inert and non-toxic (Moreno et al., 2011). All these factors make it a useful material for long-term cell culture and has found applications in tissue engineering for *in vitro* models (Harrington et al., 2014; Morris et al., 2014a,b; Haneef and Downes, 2015) and transplantable graft constructs (Hadjizadeh et al., 2011; Moreno et al., 2011).

Polyethylene terephthalate flakes (food grade; were procured from recycled plastic bottles and processed in-house) were dissolved in a 1:1 solution of dichloromethane and trifluoroacetic acid (overnight with stirring) and the polymer solution transferred into a syringe fitted with a blunt needle (18 gauge spinneret) attached to a syringe pump (Harvard Apparatus, United Kingdom). Electrospinning was conducted at a flow rate of 0.5 mL/hr at 14 kV voltage (Glassman High Voltage Inc., Series EL, Glassman Europe Ltd., United Kingdom; Supplementary Figure S1). The fibers were collected on a grounded static plate or rotating collector (a tip-collector distance of 15 cm). The scaffolds were removed with the aid of 70% (*v/v*) IMS and allowed to air dry at room temperature (RT).

The nanofibrous scaffolds were surface coated with type I bovine collagen (Sigma-Aldrich). For type I bovine collagen coating, 150 μL of collagen solution (stock and working concentrations 3.1 mg/mL and 31 μg/mL, respectively) was gently pipetted ensuring adequate coverage of the entire scaffold. Samples were incubated at RT under aseptic conditions for 1–2 h. Any remaining collagen solution was aspirated and the scaffolds washed with PBS before cell seeding. Control scaffolds included PET nanofibers with no surface treatment.

### Scaffold Characterisation

#### Scanning Electron Microscopy (SEM)

For acellular Transwell^®^ and nanofibrous scaffolds, samples were sputter coated (Leica EMSCD005) with a thin gold layer for 4–5 min prior to microscopy. Biological samples were fixed in 3% (*v/v*) glutaraldehyde in 0.1 M phosphate buffer pH 7.2 overnight at 4°C, washed with PBS (x3) and dehydrated through a series of ethanol concentrations (25–100% *v/v* ethanol in dH_2_O) for 10 min in each. Following dehydration, hexamethyldisilazane (HMDS) was added to chemically dry the samples, removed and allowed to air dry overnight at RT before sputter coating. Scanning electron microscopy (SEM) was carried out using a JEOL JSM-6100 scanning electron microscope (JEOL, United Kingdom). Where necessary, images were processed using ImageJ software (W. Rasband, National Institute of Health, United States) taking 60 measurements from 3 independently produced scaffolds. Inter-fiber distances were used as a measure of pore size when comparing the differences in nanofibrous and microfibrous PET scaffolds.

#### Wettability of the Scaffold Surface

Water contact angle (WCA) was measured using a contact angle optical tensiometer (CAM 200, KSV Instruments). A single, 10 μL drop of 18.2 MΩ de-ionized water (MilliQ water) was dispensed onto the surface and WCAs were recorded from the average of left and right contact angles from 10 frames per sample.

#### Time of Flight Secondary Ion Mass Spectrometry (ToF-SIMS) Analysis of Surface Treated Scaffolds

Effective surface adsorption of collagen was investigated by examining the difference in collagen deposition on the nanofibrous scaffolds by ToF-SIMs (ToF-SIMS IV, ION-TOF GmbH, Münster, Germany) with and without washing steps (using 18.2 MΩ de-ionized MilliQ water) and compared to untreated PET scaffolds. ToF-SIMS was conducted using a TOF-SIMS IV from ION-TOF GmbH. Data acquisition was courtesy of Dr. David Scurr (University of Nottingham). Secondary ion images were generated from sample spectra and normalized to the total ion count for each sample. All data were analyzed using SurfaceLab.

### Cell Culture and Assessment of Cell Viability

Caco-2 (used within 20 passages throughout the study) was obtained from the Clinical Pathology Laboratory, Queens Medical Centre, Nottingham and are available from ATCC. They were cultured in DMEM (Gibco) supplemented with 10% (*v/v*) foetal calf serum (FCS), 2 mM L-Glutamine solution, 1% (*v/v*) antibiotic/antimycotic solution (Gibco; final concentrations of 10000 units mL^-1^ penicillin G, 100 mg mL^-1^ streptomycin sulfate and 25 μgmL^-1^ amphotericin B) at 37°C, 5% CO_2_ in 95% relative humidity (RH). Cells were cultured to around 80% confluence before harvesting cells by passaging with Trypsin/EDTA (Sigma Aldrich). Cell culture media was replaced every 2 days. For the initial evaluation of the scaffold compatibility, Collagen type I and untreated nanofibrous scaffolds were mounted in 24-well Cell Crown^TM^ inserts (Scaffdex). The mounted inserts were then placed in 12-well culture plates and seeded with 50,000 cells in 200 μL per insert. An additional 1 mL culture media was added to the well of the plate which ensured the insert were adequately surrounded and bathed in culture media. Following 24 h of culture at 37°C, 5% CO_2_, the PrestoBlue viability assay was carried out according to manufacturer’s instructions. In brief, a working solution of 10% (*v/v*) was prepared from the stock PrestoBlue solution in HBSS and sterile filtered using a 0.2 μm pore filter. Samples were washed three times with pre-warmed PBS and the working solution of PrestoBlue solution applied to the upper chamber of the inserts. Following a 30 min incubation period at 37°C, 5% CO_2_ with shaking at 150 rpm, 100 μL aliquots were taken from each sample and dispensed in triplicate into a black 96-well plate. The fluorescence of the samples was determined using a plate reader (Tecan Infinite 200, Tecan, United Kingdom) at 530/590 nm (excitation/emission wavelengths). Subsequent viability assays were conducted on day 3 and day 6 of culture to provide an initial assessment of the benefit of the surface treatments on cell adhesion and proliferation. To evaluate barrier formation, Snapwell^®^ hanging inserts (6-well; Corning Life Sciences) were employed as unlike the CellCrown^TM^ inserts, the Snapwell^®^ hanging inserts provided discrete apical and basolateral chambers in a 6-well plate formate appropriate for assessing barrier formation. The existing polycarbonate membrane on the securing clip of the Snapwell^®^ insert were removed with a scalpel and the nanofiber scaffold affixed securely to the hanging insert by means of the securing clip (Supplementary Figure S1). Cells were seeded using the same cell density (178,000 cells/cm^2^) as had been previously optimized for Caco-2 Transwell^®^ cultures (Hubatsch et al., 2007). A cell suspension containing 200,000 cells in 500 μL were seeded onto the upper chamber of the Snapwell^®^ inserts and cultured for 15–21 days at 37°C, 5% CO_2_ in air prior for TEER evaluation and for permeability tests.

### Trans-Epithelial Electrical Resistance (TEER) Integrity Measurements

Epithelial barrier formation and integrity were determined by measuring the resistance to an ion current across the epithelium. TEER measurements were taken using the EVOM2 Voltohmmeter with STX2 chopstick electrodes (World Precision Instruments, Florida). Cell inserts were washed with PBS before the addition of pre-warmed HBSS. TEER (Ω cm^2^) values were taken and calculated by using Eq. 1:

(1) TEER (Ω cm2)=(Ω cell layer−Ω blank filter)×filter surface area (cm2)

### Immunocytochemistry

Cells were washed in PBS (x3) before being fixed in 3.7% (*v/v*) paraformaldehyde for 15 min and then washed in PBS and permeabilised with 0.5% (*v/v*) Triton-X for 5 min at 4°C. Samples were then blocked (to avoid non-specific antibody binding) in a 3% (*v/v*) BSA, 1% glycine (*v/v*) blocking solution for 30 min at ambient temperature. Samples were then washed and blocked further in 10% (*v/v*) mouse serum for 30 min at ambient temperature. The cells were then incubated with the primary antibody, mouse monoclonal primary tagged human anti-Zona Occludens-1 Alex Fluor 488 (ZO-1; Invitrogen Life Technologies, United Kingdom) used at the manufacturers recommended dilution (5 μg/mL) in blocking solution overnight at 4°C. Following this, the samples were washed with PBS and incubated with a nuclear stain, either DAPI/Hoechst 33342 for 5 min prior to viewing under fluorescence microscopy.

### Histology and Haematoxylin and Eosin Staining

The cell samples were fixed in 10% (*v/v*) formalin solution for 20 min. After washing in PBS, the samples were dehydrated in a series of ethanol solutions followed by xylene for 1 h (x2) and then finally in paraffin wax for 1 h (x2) in a tissue processor (Leica TP1020, Milton Keynes, United Kingdom). Sections were cut into 8 μm sections (Leica RM 2165 microtome) before mounting on glass slides in preparation for staining. Samples were paraffin-stripped in xylene, rehydrated and then stained by Harris haematoxylin and 1% (*w/v*) alcoholic eosin. Images were obtained using an inverted Nikon Eclipse TS100 microscope.

### Transmission Electron Microscopy (TEM)

Samples were fixed in 3% (*v/v*) glutaraldehyde (TAAB laboratories) in 0.1 M cacodylate buffer overnight at 4°C before post-fixation in 1% (*v/v*) aqueous osmium tetroxide in 0.05 M phosphate buffer pH 7.2 for 30 min. Samples were washed in water for 5 min before dehydration in 50% (*v/v*) ethanol for 2 min × 5 min steps. Cell scaffolds were removed from their inserts and stored in 70% (*v/v*) ethanol before further dehydration in 90 and 100% (*v/v*) ethanol and subsequently 100% (*v/v*) propylene oxide. Samples were infiltrated with araldite CY212 resin for 30 min (in a 1:3 resin:acetone mix), followed by 1 h (1:1 resin:acetone mix) and finally 3 h × 1 h in pure araldite resin. Samples were then embedded in resin for at least 48 h at 60°C. Ultra-thin (90 nm) sections were cut using a diamond knife and were negatively stained with uranyl acetate and lead citrate. The sections were then viewed using an FEI Tecnai G2 TEM. Image acquisition was courtesy of Denise McClean of the School of Life Sciences Imaging (SLIM), University of Nottingham Medical School.

### Porcine Intestinal Segment Preparation

In accordance with the NC3R framework on the reduction and refinement of animals in research; samples were only obtained from healthy adult pig cadavers being used for the purposes of other research projects. The work was conducted according to United Kingdom Home Office guidelines (Animal Scientific Procedures Act 2010) and following local AWERB approval (ref 000057). Regional sections from the duodenum (first 25 cm from the stomach), jejunum (150 cm from the stomach) and ileum (first 50 cm back from the ileocecal valve) were resected from pig cadavers (Supplementary Figure S2). Using tweezers the serosa and muscularis propria (muscle layers) were carefully peeled away leaving the mucosa. Sections were washed in PBS, cut to fit the Transwell^®^ inserts (Corning Life Sciences) and transferred in 6-well plates. All tissue was kept on the ice and used within a 24 h time period, post-collection.

### Drug/Marker Permeability Across the Epithelial Barrier

Transport studies were conducted using standard, widely accepted protocols as previously described (Volpe, 2008). Transport markers were prepared in transport buffer (HBSS, 25 mM HEPES, pH 7.4). Dimethyl sulfoxide (DMSO) was used to aid solubilisation of poorly soluble drugs at a maximum final concentration of 0.1% (*v/v*) used in all transport experiments. TEER was measured before and after each experiment to ensure barrier integrity during the experiments and as a control measure of experimental validity. Inserts were transferred to fresh plates containing transport buffer (apical/basolateral volumes were 0.4/1.2 mL and 0.5/6 mL for Transwell^®^ and Snapwell^®^ inserts, respectively). Donor solutions were added to the relevant compartments and the inserts were then incubated at 37°C with shaking at 150 rpm and sampled at intervals of 30 min or 1 h for 1–2 h. Apparent permeability coefficients (Papp) were calculated according to Eq. 2:

(2) $$\begin{array}{c} \mathrm{Papp}=(\mathrm{dQ}/\mathrm{dt})(1/AC0) \end{array}$$

Where *dQ*/*dt* is the flux of the compound being investigated (μM s-1), A is the exposed surface area of the insert/scaffold (cm^2^) and C0 is the concentration in the apical compartment at *t* = 0 (μM).

Compound recovery following permeability assessment were calculated according the following Eq. 3:

(3) $$\begin{array}{c} \mathrm{Recovery}(\%)=[C_{R(\mathrm{final})}\times V_{R(\mathrm{final})})\times 100/(C_{D(\mathrm{initial})}\times V_{D(\mathrm{initial})}] \end{array}$$

Where C_D_ and C_R_ are the donor and receiver compartment concentrations, V_D_ and V_R_ are the donor and receiver compartment volumes at *t* = 0 (initial) or the end of the experiment (final).

Atenolol and propranolol (Sigma Aldrich) were dissolved in a minimal volume of DMSO before dilution into transport buffer. Final DMSO concentrations were less than 0.1% (*v/v*) and donor drug/compound concentrations used for all experiments were; atenolol 1 mM, propranolol 0.5 mM, lucifer yellow (Biotium) 50 μM, FITC-dextran 4 (Sigma Aldrich) and rhodamine 123 (Biotium) 10 μM. Samples were quantified by reverse phase-HPLC with UV detection (atenolol, propranolol, and verapamil) or using a plate reader (lucifer yellow, FITC-dextran 4000 and rhodamine 123). All HPLC was performed using a Kinetix XB-C18 5 μm, 100 Å pore size, 150 mm × 4.56 mm column (with a guard column) on an Agilent 1100 HPLC system. Atenolol and propranolol were analyzed at 225 nm and 290 nm, respectively. Samples were analyzed using gradient runs with Milli-Q dH2O, 0.5% TFA (eluent A) and acetonitrile, 0.5% TFA (eluent B).

### Statistical Analysis

All statistical analysis was carried out using Graphpad Prism 6 software. Where three or more sets were compared, data were analyzed by One Way ANOVA with specific post-tests. All the measured values were expressed as mean ± standard deviation (SD) and the number of independent experiments (*n*) is noted in the figure captions.

## Results and Discussion

### Fibrous Scaffold Characterisation and Optimisation for Epithelial Cell Culture

#### Physical and Chemical Properties of Nanofibers

**FIGURE 1:**
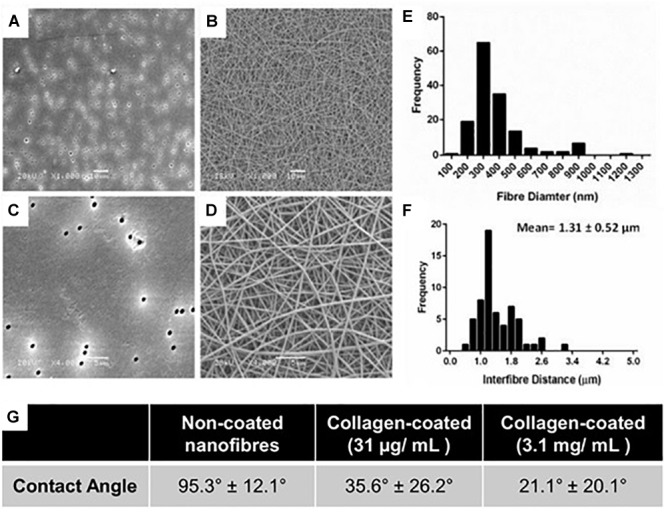
**(A,C)** High and low magnification scanning electron micrographs of conventional Transwell^®^ PET membrane inserts (pore size 0.4 μm) and **(B,D)** nanofibrous scaffolds produced by electrospinning 10% w/v PET; **A,B** scale bars = 10 μm; **(C,D)** scale bars = 5 μm. **(E)** Fiber diameter frequency distributions of scaffolds produced by electrospinning. **(F)** Frequency plot of interfibre distances for PET nanofibers produced by electrospinning. All fiber diameter data collected from *n* = 60 measurements from 3 independently produced scaffolds. **(G)** Water contact angle of untreated and collagen type I coated PET scaffolds.

Uniform fibers were fabricated by electrospinning PET (Figure 1) providing a more porous structure as compared with the commercial Transwell^®^ insert (Figure 1A,C). The fiber diameter could be modulated by altering the polymer solution concentration and flow rate. Microfibres were fabricated at polymer concentrations of 20 and 30% (w/v) with average fiber diameters of 1.1 (±0.4) μm and 2.5 (±0.4) μm, respectively (Supplementary Figure S3). Nanofibers could be produced at 10% (w/v), using a lower flow rate of 0.5 mL/h, with average fibers of 0.5 (±0.2) μm in diameter (Figure 1B,D,E). The 30% (w/v) polymer solutions exhibited thicker fibers with larger inter-fiber pore sizes measured by analysis of scanning electron micrographs. Microfibrous scaffolds had a mean inter-fiber distance of 15.8 (±8.0) μm compared to 1.3 (±0.5) μm for the nanofibrous scaffolds (Figure 1F). The fibers produced here are consistent with those reported by others. Hadjizadeh et al. (2011) reported the fabrication of PET nanofibers with an average fiber diameter of 400 nm at 7.7% polymer concentration (w/v) and 700 nm at 9% (w/v). In addition, Morris et al. (2014a) were able to produce fibers of 250 nm diameter at 8% (w/v). The capacity to fabricate and modulate nanofibers that closely mimic the dimensions of natural ECM has made electrospinning an attractive approach for conducting 3D cell culture (Hajiali et al., 2011). The compact and decreased porosity of the nanofibrous scaffold provides high cell-scaffold interaction which can be useful for cells with barrier properties, whilst an increase in fiber diameter can enhance scaffold porosity and cell infiltration (Morris et al., 2014a). As the basement membrane is a supportive structure which physically separates the epithelial cell layer from the underlying stroma, it was important that the scaffold would be suitable for this application and therefore the nanofibers made from the 10% (w/v) polymer solution were selected for further study.

**FIGURE 2:**
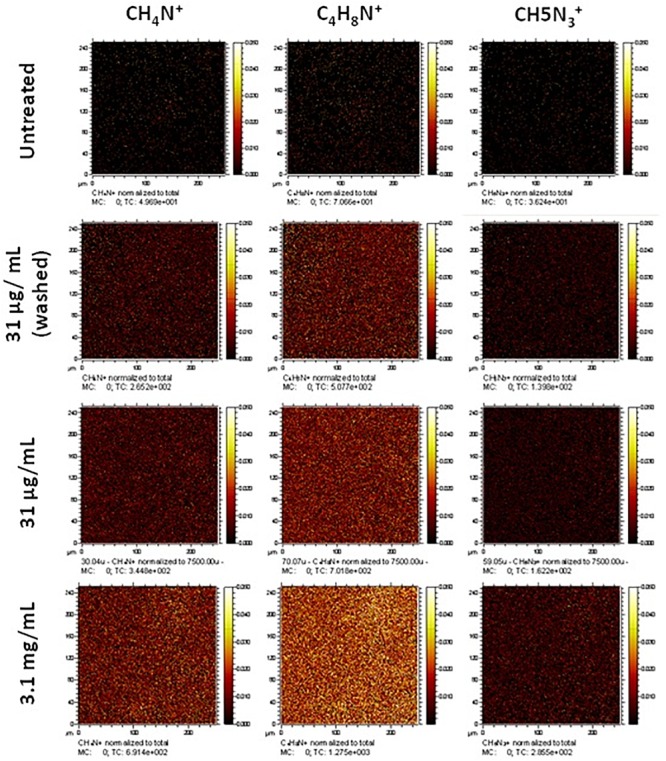
Normalized secondary ion images of untreated and collagen type I treated PET scaffolds. Ion images for unique nitrogen-containing peaks in untreated and type I collagen treated nanofibrous scaffolds. Images have been normalized to the total ion counts. Images are 250 μm × 250 μm.

Collagen treatment of the scaffolds was characterized via its impact on surface wettability by measuring the WCA of the treated scaffold at different coating concentrations compared to untreated scaffolds (Figure 1G). The untreated scaffolds were hydrophobic in nature (95.3°± 12.1°) before surface coating with collagen, 35.6°± 26.2° and 21.1°± 20.1° at 31 μg/mL (the final working concentration used) and 3.1 mg/mL (the stock concentration), respectively. The adsorption of collagen to the surface of the nanofibrous PET was investigated further by the surface analysis technique, ToF-SIMS. Furthermore, the extent of collagen remaining on the scaffolds after a wash step was studied as a measure of how strongly adsorbed the collagen was to the surface of the scaffolds. The presence of nitrogen-containing ion fragments, some of which have been attributed to specific amino acids based on literature reports (Wagner and Castner, 2001), were used as indicators of the presence of collagen. The secondary ion images of representative nitrogen-containing ion fragments generated from spectral data demonstrate that the collagen coats the surface of the scaffold in a homogenous fashion for all the treated samples (Figure 2).

Treatment at a high concentration of collagen (3.1 mg/mL) displays greater coverage of the surface with nitrogen-containing ion fragments, specifically C_4_H_8_N^+^, which is attributed to the amino acid proline in the collagen. Rasi Ghaemi et al. (2016) also analyzed immobilized Collagen I by ToF SIMS, and showed that C_4_H_8_N^+^ and CH_4_N^+^ can be used to confirm the presence of collagen on the surface. Comparable surface coverage can be observed for all three representative ion fragments but at lesser intensities in all treated samples. The sample treated at the final working concentration (31 μg/mL) that underwent a washing step (to confirm that the collagen would remain during cell culture) still showed a homogenous coating with regard to the ion fragment distribution albeit at a lower intensity. In addition to the representative secondary ion images in Figure 2, other unique nitrogen containing peaks were identified and are listed in Supplementary Table S1.

#### Examination of Cell Growth on Surface Treated of Nanofibrous Scaffolds

**FIGURE 3:**
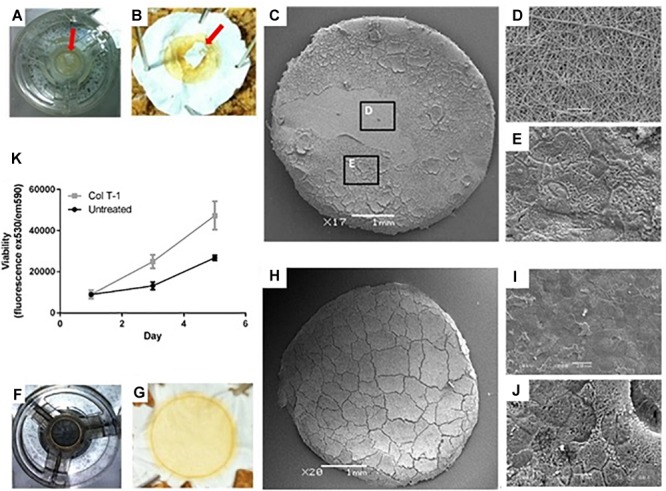
Examining the growth of Caco-2 cells on the surface of untreated and treated nanofibrous PET scaffolds. **(A,B)** Samples were fixed by 3% (*v/v*) glutaraldehyde and osmium tetroxide revealing ‘patchy’ cell growth on some scaffolds (red arrows). **(C–E)** Higher magnification SEM images showed **(D)** regions of the cell growth area devoid of cells as well as **(E)** areas covered by confluent epithelial sheets. **(F,G)** Collagen type I treated samples were fixed in 3% (*v/v*) glutaraldehyde and post-fixed with 1% (*v/v*) Osmium tetroxide illustrating the complete coverage of the scaffolds surface with cells. **(H–J)** Further SEM analysis confirmed confluent coverage of the PET nanofibrous scaffold by a sheet of Caco-2 cells. **(K)** Cell viability determined using the PrestoBlue assay of Caco-2 cultured on untreated and collagen type I treated (Col T-1) nanofibrous scaffolds. Values expressed as mean ± SD, *n* = 3.

Caco-2 culture on the untreated PET nanofibrous scaffolds demonstrated some inconsistent growth across the surface of the scaffold where confluence of the epithelial cell layer was not uniform (Figure 3A–E). Following this finding, surface treatment of the nanofibrous scaffolds was investigated to improve cell attachment and growth and to ensure complete confluence in epithelial cell growth. Initial cell attachment to the scaffolds (24 h after seeding) was the same on all scaffold types (Figure 3K). However, cells cultured on the collagen type I treated scaffolds displayed an increased cell viability compared to non-treated scaffolds in the following days of culture. Collagen coating was, therefore employed for all subsequent cell culture experiments (Figure 3F–J).

Cell confluency and sustaining barrier integrity are important factors in the design of *in vitro* epithelial models. Even during epithelial cell shedding *in vivo*, neighboring epithelial cells orchestrate redistribution of tight junctions around the shredded cell to maintain barrier integrity (Guan et al., 2011; Williams et al., 2014). Thus, ECM proteins (such as collagen, fibronectin, and laminin) provide an attachment framework for the adhesion and growth of epithelial cells *in vivo*. These adhesion molecules have been extensively used for cell attachment to various substrates *in vitro* (Harnett et al., 2007). It has been previously demonstrated that not only cell adhesion enhanced in the presence of serum proteins adsorbed on the membranes, but also their metabolic function improved on hydrophilic membranes (De Bartolo et al., 2002). Furthermore, Harnett et al. (2007) showed that collagen coatings can change the surface energy of substrates and provide hydrophobic or hydrophilic surfaces, depending on the underlying substrates. Generally, hydrophilic substrates are more favorable for cell adhesion although surface topography can also influence cell attachment (Hajiali et al., 2018).

### Evaluation of Caco-2 Barrier Formation

**FIGURE 4:**
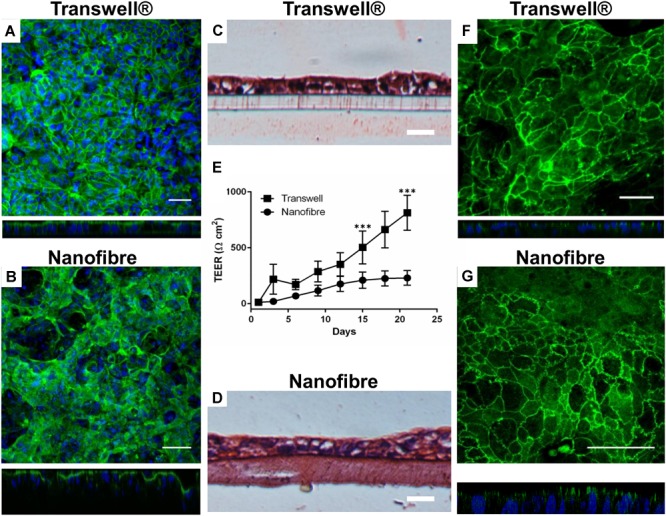
Epithelial barrier properties for Caco-2 cultures on nanofibrous scaffolds compared to Transwell^®^ based cultures. **(A,B)** Caco-2 cultures on Transwell^®^ and nanofiber supports, respectively, stained for cytoskeletal filament F-actin viewed top down and respective XZ cross sectional views; scale bars = 50 μm. **(C,D)** H&E stained Caco-2 cultures on Transwell^®^ and nanofiber supports, respectively; scale bars = 25 μm. **(E)** Trans-epithelial electrical resistance of Caco-2 cultured on Transwell^®^ and nanofiber scaffolds. Values expressed as mean ± 1 SD; *n* = 9–15 inserts from a minimum of 3 independent experiments; *P*-values: ^∗∗∗^*P* < 0.001. **(F,G)** Immunostained tight junction protein ZO-1 with XZ confocal stack cross sections, on Transwell^®^ and nanofiber supports, respectively; scale bars = 50 μm.

Caco-2 cultures (both on Transwell^®^ and nanofibrous scaffolds) were investigated to confirm the formation of an epithelial barrier and to confirm key features of a functional epithelium (Figure 4). F-actin immunostained cultures showed a confluent epithelial sheet on both the Transwell^®^ and nanofiber culture supports (Figure 4A,B). The histological cross sections displayed comparable cellular arrangement and morphology of Caco-2 cultures irrelevant of the culture substrate (Figure 4C,D). As seen with immunostaining, cellular multi-layering was evident on the nanofibrous scaffolds, characteristic of the Caco-2 cell line when differentiated to the small intestinal phenotype, which was observed but to a lesser extent on the Transwell^®^ inserts. The multi-layering of epithelial cells which are cultured *in vitro* has been reported (Madden et al., 2018).

The barrier integrity of the nanofiber matrix was assessed by measuring the increase in TEER over a 21 day culture differentiation period (Figure 4E). The Caco-2 cells cultured on Transwell^®^ supports demonstrated high final TEER readings (>500 Ω cm^2^ from day 15 onward). In these cultures, TEER dramatically increased as early as 3 days after cell seeding to around 200 Ω cm^2^ (Figure 4E) increasing exponentially. When Caco-2 cells were cultured on nanofibrous scaffolds, TEER increased early in culture at around day 6 with cultures displaying a resistance of ∼70 Ω cm^2^. Subsequently, TEER steadily increased to 200 Ω cm^2^ over the next 9 days (day 15) and finally reached a plateau between day 15 to day 21 (220 Ω cm^2^).

**FIGURE 5:**
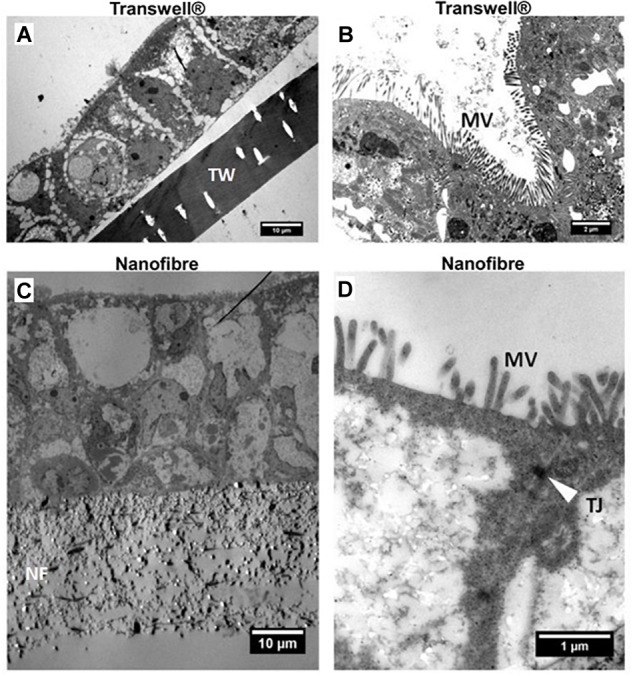
Representative transmission electron micrographs (TEM) of Caco-2 cultures. **(A,C)** Low and **(B,D)** high magnification micrographs of Caco-2 cultures on Transwell^®^ (TW) and nanofibrous (NF) scaffolds, respectively. The location of the scaffolds, microvilli (MV) and apical tight junctions (TJ – white arrow) have been highlighted.

The expression of the key tight junction protein ZO-1 (Figure 4F,G) identified distinct apical staining (specific to apical tight junctions) in addition to the typical cobblestone-like appearance of the epithelium on both substrates. Furthermore, the morphology of the cells was investigated by TEM (Figure 5). The comparative cross sectional TEM analysis showed that the cells expressed characteristic features of differentiation, such as microvilli (MV) and tight junction complexes (TJ), when cultured on both substrates.

**FIGURE 6:**
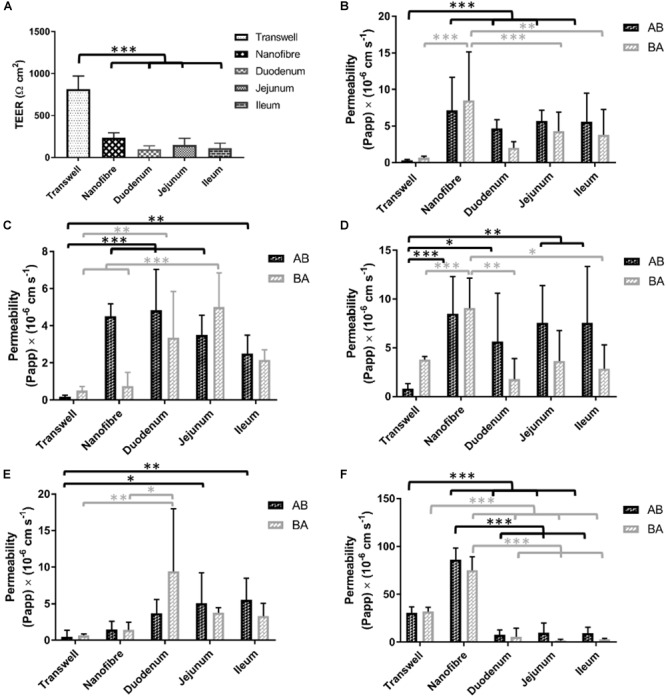
**(A)** Electrical resistance of regional porcine segments in comparison to Transwell^®^ Caco-2 cultures and nanofibrous scaffold Caco-2 culture (on day 21 of culture). **(B–F)** The bidirectional apparent permeability coefficients (Papp) of **(B)** lucifer yellow (LY), **(C)** FITC-dextran 4000 **(D)** P-gp substrate rhodamine 123, **(E)** atenolol (ATN), and **(F)** propranolol (PRN). The permeability of materials in absorptive and secretory transport directions was investigated in *in vitro* models and compared with *ex vivo* porcine tissue sections; values represent mean ± SD, *n* = 9–15 (inserts) from a minimum of two independent experiments for Transwell^®^/nanofiber models, *n* = 2–4 inserts from 2 donor pigs with respect to porcine tissue samples; *P*-values: (^∗^*P* < 0.05; ^∗∗^*P* < 0.01; ^∗∗∗^*P* < 0.001).

Porcine intestinal segments were evaluated for their TEER with respect to regional variation throughout the intestinal tract. Mean TEER values recorded were 98 (±40), 145 (±80), and 113 (±63) Ω cm^2^ (±SD) with significant differences observed between the jejunum and both the duodenum and ileum (Figure 6A). The use of resected porcine tissue has recently been developed as a more predictive tool in drug permeability assessment (Westerhout et al., 2014). Westerhout et al. (2014) reported TEER values of jejunal segments of 58 ± 7 Ω cm^2^ (generally ≤100 Ω cm^2^ is in agreement with other literature values; Moeser et al., 2007) which is in relatively good agreement with our findings (97–145 Ω cm^2^). Day 21 TEER readings from both Transwell^®^ and nanofiber culture supports were compared with the porcine intestinal segments. Caco-2 Transwell^®^ cultures exhibit a comparatively high TEER compared to cells cultured on the nanofibrous scaffolds which show electrical resistance values closer to the *ex vivo* porcine tissue sections (Figure 6A). The *in vivo* human TEER value for small intestine and colon has been reported 50–100 Ω cm^2^ and 300–400Ω cm^2^, respectively (Srinivasan et al., 2015).

### Permeability Studies

#### Lucifer Yellow (LY)

The permeability of lucifer yellow is used as a method to evaluate the integrity of Caco-2 monolayers and is used as a low permeability marker of the paracellular route of absorption (Konsoula and Barile, 2005). The permeability of this molecule is passive (i.e., not carrier/transporter mediated) and therefore the transport would be expected to be equal in both directions (apical to basolateral A to B; and basolateral to apical B to A). Caco-2 monolayers on Transwell^®^ displayed a low flux of LY with permeability coefficient values (Papp) of 0.30 (±0.13)/0.66 (±0.23) × 10^-6^ cm s^-1^ (A to B/B to A) (Figure 6B). The cultures on the nanofiber scaffolds exhibited Papp values of 7.14 (±0.4.53)/8.51 (±6.61) which were significantly higher than Transwell^®^ Caco-2 cultures. The porcine intestinal segments also displayed significantly higher permeability of LY than Transwell^®^-based Caco-2 equivalents. LY Papp values for duodenal, jejunal and ileal sections were 4.66 (±1.19)/2.01 (±0.85), 5.71 (±1.50)/4.31 (±2.58), and 5.58 (±3.93)/3.80 (±3.46) × 10^-6^ cm s^-1^, respectively. These permeability values were not significantly different from those obtained with Caco-2 cultures on the nanofibers.

#### FITC-Dextran 4000 (FD4)

FITC-dextran 4000 is a high molecular weight florescent molecule which, similar to lucifer yellow, is used to assess passive epithelial transport (Konsoula and Barile, 2005). The Transwell^®^ based Caco-2 monolayers displayed a very low Papp for this type of molecule with permeability coefficients of 0.18 (±0.10)/0.51 (±0.25) × 10^-6^ cm s^-1^ (A to B/B to A; Figure 6C). These values equate to a total molecular flux of less than 0.1%. Caco-2 cultures on nanofibrous scaffolds showed some evidence of directional transport bias with considerably increased permeability in the absorptive direction (i.e., A to B) over the secretory direction (i.e., B to A). Papp values for the nanofiber Caco-2 cultures were 4.51 (±0.67)/0.75 (±0.74) × 10^-6^ cm s^-1^ with the absorptive permeability being significantly higher than that of the Transwell^®^ based cultures. Porcine segments all displayed FD4 Papps between 2.5–4.8 × 10^-6^ cm s^-1^ (AB direction) and 1.85–5.00 × 10^-6^ cm s^-1^ (BA direction). Again, the permeability coefficients achieved with the *ex vivo* porcine tissues were comparable to the nanofiber Caco-2 cultures and significantly higher than Transwell^®^ based cultures. FD4 recovery for porcine intestinal segments was similar to LY with recoveries ranging from 50–70%.

#### Rhodamine 123 (Rho 123)

Rhodamine is a known substrate of p-glycoprotein (P-gp/MDR1) and exhibits active efflux which can be demonstrated *in vitro* with the Caco-2 model (Troutman and Thakker, 2003). The Transwell^®^ based Caco-2 cultures exhibited this mode of transport with Rho-123 Papp values of 0.82 (±0.51)/3.80 (±0.31) × 10^-6^ cm s^-1^ (A to B/B to A; Figure 6D). These values give an efflux ratio of 4.6 which is indicative of active efflux (Hubatsch et al., 2007). Despite being able to observe this efflux phenomenon in the Transwell^®^ cultures, this was not detectable in the 3D nanofiber based cultures nor in the porcine tissue segments. Rho-123 Papp in porcine segments ranged from 5.6–7.6 × 10^-6^ cm s^-1^ for A to B transport and 1.8–3.6 × 10^-6^ cm s^-1^ B to A permeability. Compound recovery was notably poor for Rho-123 with recoveries between 37 and 54%. Nanofiber based cell cultures showed increased permeability over the porcine tissue segments and, similar to the porcine sections, did not demonstrate the directional bias toward the efflux pathway but rather showed relatively equal permeability in both directions.

#### Atenolol (ATN)

Atenolol (ATN) is a commonly utilized drug for Caco-2 based assessment of permeability due to the fact it has been widely used in *in vivo* studies and there is general agreement upon the fraction absorbed (%FA) being roughly 50% of the administered dose. As such it helps serve as one of several compounds regularly used to provide a rank order of drugs with known FA values which contributes to permeability predictions for novel compounds using the Caco-2 model (Volpe et al., 2007). Atenolol permeates mainly via the paracellular route of absorption similar to LY and FD4. Typically low permeability’s are observed in Caco-2 monolayers (Volpe et al., 2007), in this study baseline Caco-2 Papp values (AB/BA) for ATN were 0.48 (±0.09)/0.67 (±0.21) × 10^-6^cm s^-1^ (Figure 6E). The nanofiber based cultures showed increased permeability to atenolol [1.48 (±1.32)/1.44 (±1.04) × 10^-6^ cm s^-1^] over the Transwell^®^ Caco-2 systems, but the differences were not significant. The porcine intestinal segment permeability ranged from 3.71–5.58 (A/B) and 3.22–9.44 (B/A) × 10^-6^ cm s^-1^ with an average recovery of 80% (range between 70 and 95%). As with the majority of the other tested compounds, no significant gastrointestinal regional variation was observed in ATN permeability, in either transport direction. The Papp values were however, significantly higher than Caco-2 Transwell^®^ culture for jejunal and ileal segments in the AB direction (5.08 ± 4.12 × 10^-6^ cm s^-1^ and 5.52 ± 2.97 × 10^-6^cm s^-1^) but only for duodenal tissue in the BA direction (9.44 ± 8.56 × 10^-6^cm s^-1^).

#### Propranolol (PRN)

Propranolol (PRN), similar to atenolol, is a commonly used compound to probe the permeability properties of *in vitro* models with a well-known FA (>90%). Due to the lipophilic properties of PRN, passive transcellular permeability is the dominant mechanism of permeability across the epithelium (Lennernäs et al., 1997). Propranolol demonstrated high permeability in the Transwell^®^ Caco-2 cultures 30.67 (±6.32)/32.03 (±4.41) × 10^-6^ cm s^-1^ (AB/BA) with consistency in flux in both directions. In the nanofiber based culture system, PRN displayed vastly higher transport flux than in the Transwell^®^ cultures demonstrating more than a two-fold increase in PRN permeability [86.34 (±12.14)/75.14 (±14.23) × 10^-6^ cm s^-1^; Figure 6F]. The porcine tissue segments exhibited very low permeability coefficients 7.76–9.6 × 10^-6^ cm s^-1^ in the A to B direction and 1.85–5.50 × 10^-6^ cm s^-1^ in the B to A direction with a lower recovery with an average of 58% (range between 35 and 75%).

It is notable that the Caco-2 cultures on the 3D nanofiber scaffolds displayed lower TEER values than the Transwell^®^ equivalents which potentially corroborates the observed increase in molecular flux. Despite these lower TEER values, the typical cellular morphology with microvilli and apical tight junction formation, in addition to ZO-1 staining indicates functional tight junction expression. The mechanism of the decrease in TEER has not been clarified in detail but previous work from our group suggests that it would be a direct result of the increased porosity of nanofibers over the Transwell^®^ inserts (Morris et al., 2014a). In addition, the other plausible cause of this is a decreased expression of tight junction proteins under 3D culture conditions (Li et al., 2013). Yu et al. (2012) also observed a significantly lower TEER value in a 3D model than those obtained using 2D culture. However, a detailed molecular pathway underlying these similar results across several different studies has not yet been identified. We propose here that the results of our study demonstrate that the structural organization of gut epithelium in the nanofiber model influences the physiology of Caco-2 cells in such a way that a more *in vivo*-like barrier function is observed.

To the authors’ knowledge, the data presented herein is the first extensive investigation into the permeability properties of Caco-2 and nanofiber based culture systems as alternative platforms for predicting drug permeability *in vitro.* In terms of molecular permeability, our results from the rhodamine-123 permeability experiments (where the efflux ratio of Rho 123 in the Transwell^®^ was greater than that in nanofibers and tissue segments) propose that P-gp is highly active in the Transwell^®^ model compared to when cells are cultures on the nanofibers and in porcine tissue segments. A study by Yi et al. (2017) evaluated the efflux ratio of rhodamine-123 in 2D and 3D models, and they also showed a decreased efflux ratio of Rho 123 in the 3D model. In addition, Li et al. (2013) studied the efflux of digoxin, which is also a substrate of P-gp and demonstrated a reduction in the efflux permeability of digoxin in the 3D model. The multi-drug resistance (MDR1) gene encodes an efflux transporter P-gp, and it has been demonstrated that the expression levels of MDR1 in Caco-2 was 2-fold higher than that of human jejunum (Harwood et al., 2016). Whilst P-gp functionality was not explored, the mentioned study suggests Caco-2 cells overexpress MDR1 and thus most likely over exaggerates efflux compared to the *in vivo* situation. Furthermore, our data suggested that the P-gp was either not active or its function was masked in the *ex vivo* porcine tissue segments. Expression of P-gp in this model system warrants further investigation but was not the focus of our work here.

Westerhout et al. (2014) reported Papps for atenolol and propranolol of 1.25/2.92 × 10^-6^ cm s^-1^ (in mini pigs/normal pigs) and 0.42/25.1 × 10^-6^ cm s^-1^ (Papp/Papp_total_), respectively. The low Papp value for propranolol was attributed to the poor compound recovery for which the authors corrected for by factoring in tissue accumulation (Papp_tissue_) (Rozehnal et al., 2012). Tissue accumulation is likely to be significant for highly lipophilic compounds such as propranolol (see Papp_total_ value for corrected value which is 60-fold higher than the Papp). However, a similar correction could not be applied to our data sets due to the experimental design. Despite this, the calculated Papps for LY and ATN demonstrated a correlation with reported human jejunal Papp values by Sjöberg et al. (2013) and by Rozehnal et al. (2012) (4.02 ± 2.20 and 5.56 ± 2.21 × 10^-6^ cm s^-1^ for LY and mannitol, respectively and 4.11 ± 1.32 and 2.82 ± 0.65 × 10^-6^ for ATN).

In summary, Caco-2 cells cultured upon a nanofibrous scaffold more closely represents *ex vivo* mammalian epithelial tissue with respect to *in vitro* permeability, in particular as a model for passive epithelial transport. This model also demonstrates permeability coefficients values closer to human *ex vivo* tissue (Rozehnal et al., 2012; Sjöberg et al., 2013). Despite the widespread application of 2D Caco-2 cell models in the field of drug discovery, its true correlation with the *in vivo* situation remains in question (Balimane and Chong, 2005). For example, 2D monolayers of Caco-2 cells have been known to under predict the absorption of some compounds, namely those where paracellular transport is thought to be dominant (Balimane and Chong, 2005).

## Conclusion

The aim of this study was to develop a simple yet more *in vivo*-like *in vitro* model of the human intestinal epithelium. We evaluated the physiological properties of Caco-2 cells cultured on collagen-coated PET 3D nanofibrous scaffolds, in comparison to 2D Caco-2 cultures on a Transwell^®^ membrane and assessed barrier integrity and drug-permeability properties. Our data demonstrate that the PET 3D nanofibrous scaffold influences the barrier integrity of Caco-2 monolayer to confer drug-absorption properties that more closely mimic native gut tissue particularly for studying of passive epithelial transport. We propose therefore that this model is a more suitable *in vitro* model for investigating passive drug absorption than currently employed 2D cultures.

## Ethics Statement

In accordance with the NC3R framework on the reduction and refinement of animals in research; samples were only obtained from healthy adult pig cadavers being used for the purposes of other research projects. The work was conducted according to United Kingdom Home Office guidelines (Animal Scientific Procedures Act 2010) and following local AWERB approval (Ref 000057).

## Author Contributions

JP conducted all the laboratory based research and data interpretation. HH wrote the manuscript and organized the data into the figures presented here, led by FR. AG, PW, CR, LW, and FR conceived the project and supervised the Ph.D. student JP. KH, BA, and CT provided advice about current *in vitro* models used in the pharmaceutical industry and how to assess model compounds for the permeability studies. All authors have contributed to the manuscript and have agreed to its submission for publication.

## Conflict of Interest Statement

The authors declare that the research was conducted in collaboration with AstraZeneca PLC through a Doctoral Training Centre held at the University of Nottingham funded by the Engineering and Physical Sciences Research Council (EPSRC; studentship awarded to JP). KH, BA, and CT were employed by AstraZeneca PLC. The remaining authors declare that the research was conducted in the absence of any commercial or financial relationships that could be construed as a potential conflict of interest.
