# Supplementary material for: Nanofibrous Scaffolds Support a 3D in vitro Permeability Model of the Human Intestinal Epithelium
Source: Front Pharmacol. 2019 May 10;10:456. doi: 10.3389/fphar.2019.00456 (PMC6524416; doi:10.3389/fphar.2019.00456)
Supplement: Supplementary file 1 [file Data_Sheet_1.docx]

Supplementary Material

Nanofibrous scaffolds support a 3D in vitro permeability model of the human intestinal epithelium.

JD Patient^1$^, H Hajiali^1$^, K Harris^2^, B Abrahamsson^3^, C Tannergren^3^, L J White^1^, AM Ghaemmaghami^4^, PM Williams^1^, CJ Roberts^1^, FRAJ Rose^1*^

^1^School of Pharmacy, University Park, University of Nottingham, NG7 2RD, UK.

^2^AstraZeneca, Charter Way, Macclesfield SK10 2NA, UK.

^3^AstraZeneca, Pepparedsleden 1, 431 50 Mölndal, Sweden.

^4^School of Life Sciences, University of Nottingham, Queen’s Medical Centre Campus, NG7 2UH, UK.

^$^denotes joint first authors

*** Correspondence:**Felicity R A J Rose;
felicity.rose@nottingham.ac.uk

# Supplementary Figures and Table

**
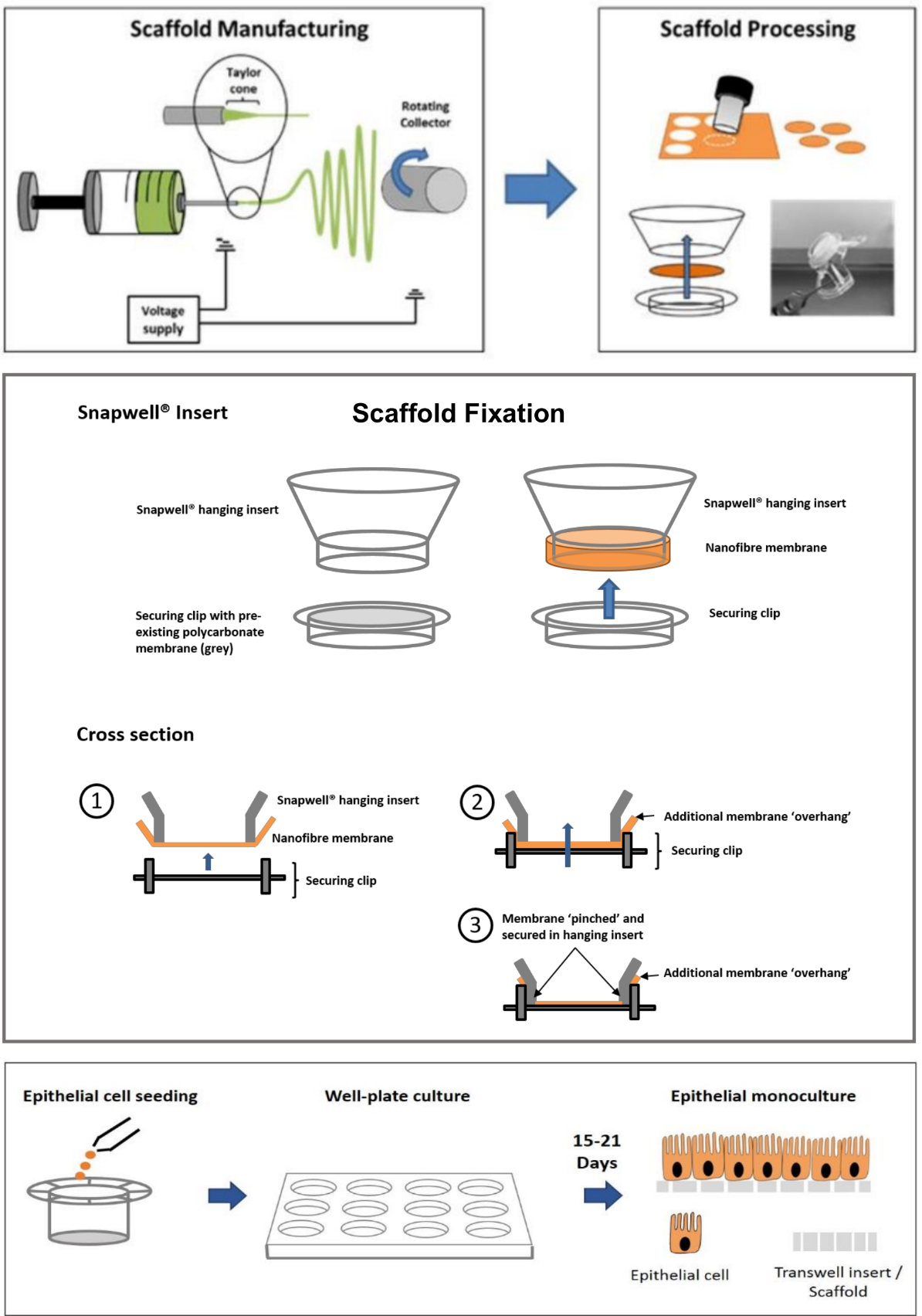
**

**Supplementary Figure 1.** Schematic work flow of scaffold production, processing, fixation, and ultimate use as topographically relevant 3D scaffolds for *in vitro* cell culture. Scaffolds produced by electrospinning can be tailored with ubiquitous hanging inserts. Epithelial cells were seeded into the upper chamber and the hanging insert suspended in a well plate. Cells were cultured for 15-21 days to allow barrier formation prior to drug permeability tests.

**
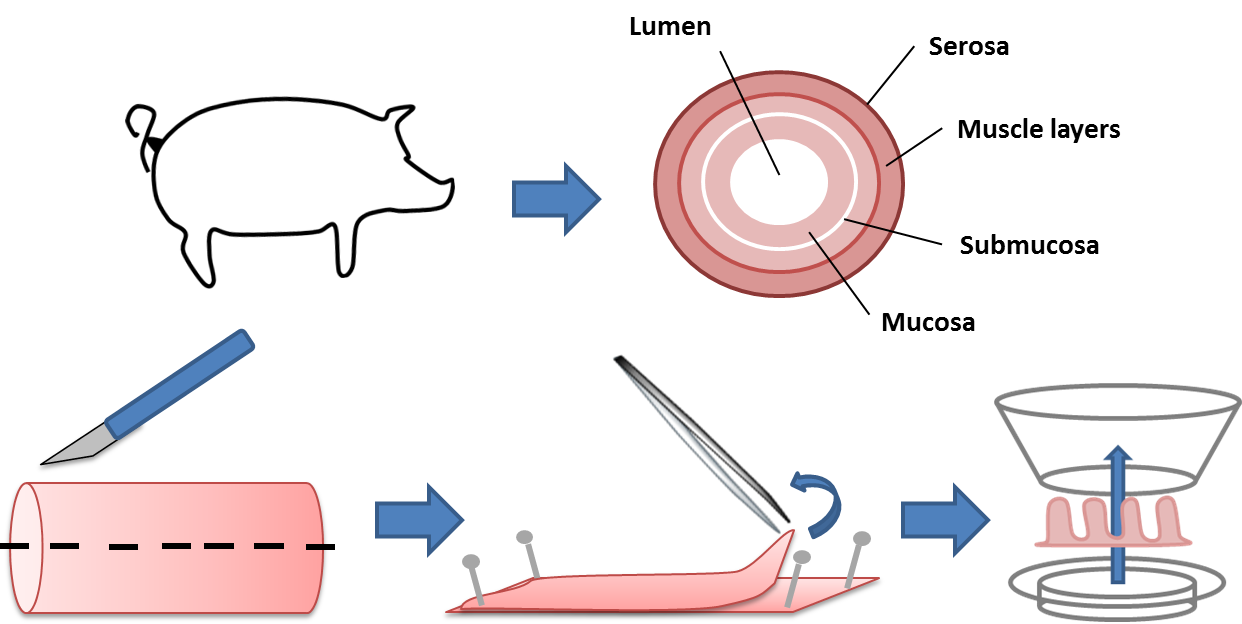
**

**Supplementary Figure 2**. Isolation and preparation of porcine intestinal segments. Intestinal sections were taken from porcine cadavers and stored in ice cold HBSS. Individual pieces were sectioned lengthways and pinned serosa side facing upwards. Using tweezers the serosa and muscularis propria were carefully peeled away leaving the mucosa. Sections were then cut appropriately to fit the Transwell® inserts and used in 6-well plates.


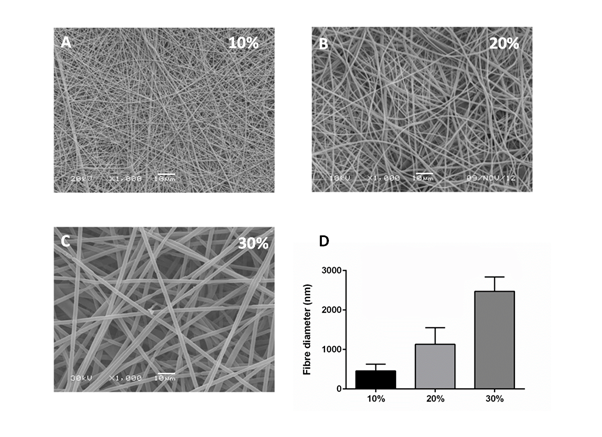


Supplementary Figure 3. PET fibre scaffold morphology and diameter analysis.

Representative scanning electron micrographs of PET fibre scaffolds produced with polymer solutions of increasing concentration; (A) 10% (*w/v*), (B) 20% (*w/v*) and (C) 30% (*w/v*). (D) Comparison of the average fibre diameters of the scaffolds at different polymer concentrations. Values are mean ± SD; data taken from total of n=80 measurements from a minimum of 3 independently produced scaffolds.

Supplementary Table 1. Positive ion peaks identified from ToF SIMs analysis of nanofibrous scaffolds.

Unique nitrogen containing peaks were identified from spectra to analyse the extent of collagen adsorption to the surface of the fibre scaffolds under different treatment conditions after a simulated ‘wash’ step.

| Source | Ion | m/z | Deviation (ppm) | | |
| --- | --- | --- | --- | --- | --- |
|  |  |  | **31 µg/mL (washed)** | **31 µg/mL** | **3.1 mg/mL** |
| Collagen | NH_3_^+^ | **17** | 18.2 | -14.3 | 12 |
|  | | | | | |
| Glycine | C_2_H_6_N^+^ | **30** | 47.7 | 48.6 | 45.9 |
|  | CH_6_N^+^ | **32** | 38.9 | 57.3 | 44 |
|  | C_2_H_6_N^+^ | **44** | 77.5 | 71.4 | 49.3 |
|  | | | | | |
| Arginine | CH_5_N_3_^+^ | **59** | 35.3 | 8.3 | 18.8 |
|  | CH_6_N_3_^+^ | **60** | 4.9 | 18.8 | 15.8 |
|  | C_4_H_6_N^+^ | **68** | 78.9 | 70.3 | 44.8 |
|  | | | | | |
| Proline | C_4_H_8_N^+^ | **70** | 81.5 | 87 | 79.2 |
|  | | | | | |
| Valine | C_4_H_10_N^+^ | **72** | -77.8 | 88 | 30 |
|  | | | | | |
| Arginine | C_2_H_7_N_3_^+^ | **73** | -60.1 | -45.9 | -35.5 |
|  | | | | | |
| Glutamic acid | C_4_H_6_NO^+^ | **84** | 29.6 | 27.8 | 30 |
